# Efficacy and safety of cilostazol in decreasing progression of cerebral white matter hyperintensities—A randomized controlled trial

**DOI:** 10.1002/trc2.12369

**Published:** 2022-12-27

**Authors:** Bonaventure Y. M. Ip, Bonnie Y. K. Lam, Vincent M. H. Hui, Lisa W. C. Au, Mandy W. T. Liu, Lin Shi, Vivian W. Y. Lee, Winnie C. W. Chu, Thomas W. Leung, Ho Ko, Vincent C. T. Mok

**Affiliations:** ^1^ Division of Neurology Department of Medicine and Therapeutics The Chinese University of Hong Kong Shatin Hong Kong SAR China; ^2^ Gerald Choa Neuroscience Institute Margaret K.L. Cheung Research Centre for Management of Parkinsonism Therese Pei Fong Chow Research Centre for Prevention of Dementia Lui Che Woo Institute of Innovative Medicine Li Ka Shing Institute of Health Science Lau Tat‐chuen Research Centre of Brain Degenerative Diseases in Chinese Faculty of Medicine The Chinese University of Hong Kong Shatin Hong Kong SAR China; ^3^ Nuffield Department of Clinical Neurosciences Wellcome Centre for Integrative Neuroimaging University of Oxford Oxford UK; ^4^ Department of Imaging and Interventional Radiology The Prince of Wale Hospital The Chinese University of Hong Kong Shatin Hong Kong SAR China; ^5^ BrainNow Research Institute Shenzhen Guangdong Province China; ^6^ Centre for Learning Enhancement and Research The Chinese University of Hong Kong Hong Kong SAR China

**Keywords:** brain, cerebral small vessel diseases, cilostazol, clinical trials, dementia, intention‐to‐treat analysis, magnetic resonance imaging, white matter

## Abstract

**Introduction:**

Cerebral small vessel disease (SVD) is an important cause of dementia that lacks effective treatment. We evaluated the efficacy and safety of cilostazol, an antiplatelet agent with potential neurovascular protective effects, in slowing the progression of white matter hyperintensities (WMHs) in stroke‐ and dementia‐free subjects harboring confluent WMH on magnetic resonance imaging (MRI).

**Methods:**

In this single‐center, randomized, double‐blind, placebo‐controlled study, we randomized stroke‐ and dementia‐free subjects with confluent WMHs to receive cilostazol or placebo for 2 years in a 1:1 ratio. The primary outcome was change in WMH volume over 2 years. Secondary outcomes were changes in brain volumes, lacunes, cerebral microbleeds, perivascular space, and alterations in white matter microstructural integrity, cognition, motor function, and mood.

**Results:**

We recruited 120 subjects from October 27, 2014, to January 21, 2019. A total of 55 subjects in the cilostazol group and 54 subjects in the control group were included for intention‐to‐treat analysis. At 2‐year follow‐up, the changes in WMH volume were not statistically different between cilostazol treatment and placebo (0.3±1.0 mL vs −0.1±0.8 mL, *p* = 0.167). Secondary outcomes, bleeding and vascular events, were also not statistically different between the two groups.

**Discussion:**

In this trial with stroke‐ and dementia‐free subjects with confluent WMHs, cilostazol did not impact WMH progression but demonstrated an acceptable safety profile. Future studies should address the treatment effects of cilostazol on subjects at different clinical stages of SVD.

## 1 BACKGROUND

Cerebral small vessel disease (SVD) contributes to almost 50% of global dementia and is associated with major disability and mortality.^1^ Despite improved understanding of SVD pathogenesis in recent years,^2^ there is a lack of effective preventive or disease‐modifying treatment for the disease beyond cardiovascular risk factor control.^3^

Because SVD is a whole‐brain, neurogliovascular disorder involving endothelial cells, astrocytes, neurons, oligodendrocytes, and secondary Wallerian degeneration,^3^ targeting these pathological processes may slow the progression of SVD. Cilostazol is a phosphodiesterase III inhibitor commonly used in the Asia‐Pacific regions for secondary stroke prophylaxis and peripheral vascular disease.^4^ In addition to antiplatelet and vasodilatory properties, cilostazol preserved endothelial function and ameliorated gliovascular damage and working memory impairment in animal studies.^5^, ^6^ Nonetheless, SVD animal models induced by chronic hypoperfusion or hypertension may not model all aspects of human SVD pathologies such as aging, multiple vascular risk factors, and complicated cerebral hemodynamics. Verification of these findings with human subjects is therefore needed. Thus far the majority of human clinical trials evaluated the effect of cilostazol on stroke recurrence,^4^ whereas data that specifically addressed cognitive and imaging outcomes were limited, with heterogeneous study populations and endpoints.^7^, ^8^, ^9^, ^10^, ^11^ Although randomized‐control trials yielded mixed results in cognitive outcomes in SVD patients with a history of lacunar strokes or intracerebral hemorrhage,^10^, ^11^ a registry‐based retrospective study showed that the use of cilostazol may reduce incident dementia.^12^ Furthermore, the benefits of treatment during the asymptomatic stage of SVD are uncertain.^13^ Due to the high prevalence of SVD in the elderly population and its prolonged indolent course,^14^ exploring interventions during the relatively quiescence phase of SVD may reveal a treatment window to prevent or delay the onset of its clinical manifestations—including stroke, cognitive impairment, dementia, and movement disorders.

We conducted the DREAM trial (efficacy and safety of cilostazol in DecREasing progression of cerebrAl white Matter hyperintensities [WMHs]) to determine the efficacy and safety of cilostazol in preventing SVD progression in stroke‐ and dementia‐free subjects with confluent WMHs—a radiological SVD marker that predicts the risk of cognitive decline and dementia.^15^, ^16^, ^17^ We hypothesized that cilostazol treatment, compared with cardiovascular risk factor control alone, would reduce WMH progression in stroke‐ and dementia‐free subjects with SVD.

## 2 METHODS

### 2.1 Trial design

The DREAM trial was a single‐center, randomized, double‐blind, placebo‐controlled, investigator‐initiated trial conducted in the Prince of Wales Hospital, a university teaching hospital in Hong Kong. We assigned stroke‐ and dementia‐free subjects with moderate‐to‐severe confluent WMH in a 1:1 ratio to either cilostazol (at a dosage of 100 mg twice daily per os) or placebo for 104 weeks using computer‐generated randomization codes. Randomization was concealed until the participants were assigned to cilostazol or placebo.

### 2.2 Study participants

We recruited participants from the prospective Chinese University of Hong Kong RISK Index for Screening Subclinical Brain Lesions in Hong Kong (CU‐RISK) cohort that screened community‐dwelling individuals with subclinical WMHs.^18^ Inclusion criteria were (1) age 65 to 85 years; (2) Chinese ethnicity; (3) beginning confluent WMHs on brain magnetic resonance imaging (MRI) in addition to an Age‐related White Matter Changes (ARWMC) global score of ≥2,^19^ as rated by neurologists or neuroradiologists with more than 10 years of experience (B.I., L.A., L.S., W.C.). Examples of the ARWMC grading of our study subjects are shown in Figure S1. Exclusion criteria were (1) history of clinical stroke or transient ischemic attack; (2) dementia according to the Diagnostic and Statistical Manual of Mental Disorders, Fourth Edition^20^; (3) peripheral arterial disease that necessitated cilostazol use; (4) concurrent use of other antiplatelet agents or anticoagulants; (5) contraindications to cilostazol (e.g., heart failure, prior history of cilostazol allergy); (6) severe medical co‐morbidities (e.g., malignancy, end‐stage renal disease, etc.); and (7) contraindications to MRI (e.g., claustrophobia, non‐MRI compatible implants, etc.). All participants received standard therapy for the management of hypertension, diabetes mellitus, and hyperlipidemia (if any) on top of trial medication.

### 2.3 Data collection

Age, sex, education level, smoking and drinking status, medications, and co‐morbidities (hypertension, diabetes mellitus, hyperlipidemia, heart disease) were recorded at baseline. In addition, we assessed blood pressure, body mass index, complete blood count, renal and liver function, fasting plasma glucose, glycosylated hemoglobin, and lipid profile (triglyceride, total cholesterol, low‐density lipoprotein cholesterol, high‐density lipoprotein cholesterol) at 52‐week intervals throughout the 104‐week study period.

**RESEARCH IN CONTEXT**

1. **Systematic review**: Some clinical studies suggested that cilostazol may improve cognitive outcomes in patients with cerebral small vessel disease (SVD) who had history of lacunar strokes or intracerebral hemorrhage. However, treatment effect of cilostazol in stroke‐ and dementia‐free patients harboring moderate‐to‐severe SVD is unknown.
2. **Interpretation**: Cilostazol treatment did not impact the progression of white matter hyperintensity volume. Our study findings did not support the routine use of cilostazol in patients with SVD without overt clinical symptoms.
3. **Future directions**: Future cilostazol trials should address treatment effects on subjects at different clinical stages of SVD.

**HIGHLIGHT**

- The efficacy and safety of cilostazol in stroke‐ and dementia‐free subjects with cerebral small vessel disease (SVD) are unclear.
- In this single‐center, randomized, double‐blind, placebo‐controlled study, cilostazol did not impact white matter hyperintensity progression on brain magnetic resonance imaging over a period of 2 years compared to placebo.
- Changes in cognition, bleeding, and vascular events were not statistically different between cilostazol and placebo treatment.
- Further studies should address treatment effects of cilostazol on subjects at different clinical stages of cerebral SVD.

### 2.4 Neuroimaging acquisition and analysis

Brain MRI was performed at baseline and 2 years. MRI was performed using the Philips Achieva 3.0 T Tx series (Philips Medical System, Best, The Netherlands). MRI sequences were acquired as follow: three‐dimensional (3D) T1‐weighted (repetition time/echo time [TR/TE]: 7.46/3.46 ms, reconstructed voxel size: 0.60 × 1.04 × 1.04 mm^3^); T2‐weighted (TR/TE: 2743.89/80 ms, reconstructed voxel size: 5.5 × 0.22 × 0.22 mm^3^); 3D fluid‐attenuated inversion recovery (FLAIR; TR/TE/TI [inversion time]: 8000/336.45/2400 ms, reconstructed voxel size: 0.55 × 0.44 × 0.44 mm^3^); diffusion‐weighted (TR/TE: 8907.32/60 ms, reconstructed voxel size: 1 × 1 × 2 mm^3^) and venous BOLD (TR/TE: 16/23 ms, reconstructed voxel size: 0.45 × 0.45 × 1 mm^3^). WMHs, lacune, cerebral microbleed (CMB), and perivascular space (PVS) were defined and visually rated according to the standards for reporting vascular changes on neuroimaging (STRIVE) criteria.^21^, ^22^, ^23^ All manual radiological assessments were performed by neurologists or neuroradiologists blinded to the time sequence, treatment allocation, and radiological assessments.

### 2.5 Baseline measurement of WMH, total brain volume, hippocampal ratio, and diffusion metrics

The T1‐weighted images were pre‐processed with bias‐field correction, brain‐extraction, and partial‐volume tissue segmentation using Functional MRI of the Brain's Automated Segmentation Tool (FAST).^24^ FLAIR images were processed with bias‐field correction using FAST.^24^ Both the bias‐corrected T1‐weighted and FLAIR images were then fed into the lesion prediction algorithm (LPA) to generate cross‐sectional lesion probability maps.^25^ The normalized WMH volume was calculated using WMH volume divided by the intracranial volume (Lesion segmentation tool version 3.0.0 for Statistical Parametric Mapping; SPM). Total brain volume, normalized for subject head size, was estimated with SIENAX.^26^ The hippocampal ratio, which was hippocampal volume divided by the intracranial volume, was quantified by Accubrain (BrainNow Medical Technology Company Ltd.), a cloud‐based automated brain quantification tool.^27^

For diffusion‐weighted imaging, the pre‐processing steps included susceptibility‐induced distortion, eddy‐current, and head motion corrections. Because the diffusion‐weighted data were acquired without reversed phase‐encoding directions, we first employed Synb0‐DISCO v2.0, a tool that synthesizes an “undistorted” b0 image through a deep learning approach (generative adversarial network), based on the geometry of the given structural T1‐weighted scans.^28^ Diffusion metrics including fractional anisotropy (FA) and mean diffusivity (MD) were acquired using DTIFIT Tract‐Based Spatial Statistics (TBSS v 1.2).^29^

### 2.6 Longitudinal neuroimaging analyses

For quantification of longitudinal changes in WMH, the automatic lesion segmentation tool (LPA) from LST, an open‐source toolbox for SPM was applied. A joint lesion map was rendered from lesions maps of both time points to identify lesion (of any time point) and non‐lesion voxels. The distribution of FLAIR intensity differences was estimated within the non‐lesion voxels to enable statistical quantification of intensity changes within the joint lesion map. Significant changes were labeled as increase or decrease in lesion volume, whereas non‐significant changes but different cross‐sectional lesion segmentation results were counted as lesions at both time points.^30^ Longitudinal changes of brain volume were measured using a modified script of SIENA. The steps include siena_nobet, which skips the brain extraction steps in the pipeline, but requires users to provide brain‐extracted and skull images (outputs from fsl_anat) from both time points to constrain the registration scaling. The subsequent procedures are identical to the original pipeline. The pre‐processed baseline and follow‐up T1 images were used to estimate the percentage brain volume change between the two time points.^26^ Longitudinal changes of white matter structural integrity were measured using the longitudinal TBSS pipeline. To avoid removing within‐subject longitudinal differences from using a different non‐linear warp on the same subject at multiple time points, we created a non‐biased, halfway‐space template for each subject.^29^

All images were visually checked and de‐identified prior to any analyses. The imaging analyses were performed mainly on the Functional MRI of the Brain Software Library (FSL) v6.0 tools^31^ and SPM12 (http://www.fil.ion.ucl.ac.uk/spm/software/spm12).

### 2.7 Clinical assessment

Assessments on cognition, mood, and motor functions were performed at baseline, 1 year, and 2 years. We assessed cognition using the Chinese Montreal Cognitive Assessment (MoCA) and the 30‐min battery of the National Institute of Neurological Diseases and Stroke – Canadian Stroke Network Vascular Cognitive Impairment (NINDS‐CSN VCI) Neuropsychology Protocol, which consists of 1‐min animal fluency for executive function, symbol digit modalities test (SDMT) for processing speed, and Hong Kong list learning test (HKLLT) for memory and verbal learning.^32^ The Chinese version of the Geriatric Depression Scale (GDS) was used to assess mood symptoms.^33^ Motor functions in walking speed and balance were assessed by 8‐m walk time and single leg stance, respectively. We measured the time taken for participants to walk 8 m twice per assessment; the faster of the two trials was used for analysis. For balance assessment, participants were asked to perform a single‐leg stance, placing hands on their hips for an upper limit of 30 seconds with two trials for each leg per assessment. The longest time of the four trials was used for analysis. All cognitive and motor assessments were performed by certified health care professionals blinded to the time sequence, treatment allocation, and radiological assessments.

### 2.8 Outcomes

The primary outcome was the absolute change in WMH volume on MRI over 2 years. Secondary imaging outcomes included changes in diffusion measures, lacunes, CMB, PVS, total brain parenchymal volume, and hippocampal volume. Secondary clinical outcomes were changes in MoCA, NINDS‐CSN VCI Neuropsychology Protocol 30‐min battery, GDS, 8‐m walk time, and single leg stance test over 2 years. Safety outcomes were intra‐ or extra‐cranial bleeding, vascular events, and death during the study period. No interim analyses were performed.

### 2.9 Study protocol, ethics approval, and patient consents

A detailed study protocol is provided in the supplementary materials. The protocol was approved by the local institutional review board (Joint CUHK‐NTEC CREC Reference No. 2013.680‐T). Written informed consent was obtained from all participants. The trial was registered with the Center for Clinical Research and Biostatistics, the Chinese University of Hong Kong (CUHK‐CCT00430).

### 2.10 Sample size estimation

Sample size estimation was based on the data from the VITATOPS MRI substudy.^34^ The median (interquartile range) of WMH volume change over 2 years for subjects with pure severe SVD was 0.04 (0–14.7) cm^3^ in the placebo group and 1.7 (0–13.6) cm^3^ in the treatment group. Assuming the effect of cilostazol upon WMH progression in subjects with confluent WMH over a 2‐year period was comparable to that of B vitamins, to detect a 1.66 cm^3^ difference in median WMH volume change with 80% power, the sample size required for each of the 2 groups was 53. Based on our experience from VITATOPS MRI sub‐study and previous trials using cilostazol among stroke subjects, we estimated that about 20% of subjects with confluent WMH may drop out over a study period of 2 years. Hence, this study required 70 subjects per group, that is, a total of 140 subjects. Recruitment of this study was prematurely stopped in 2019 due to the social unrest in 2019.

### 2.11 Statistical analyses

Continuous variables were expressed as mean ± SD, whereas categorical variables were expressed in number (percentage). We compared baseline characteristics between the two treatment groups by chi‐square or Fisher exact test for categorical variables and independent *t*‐test or Mann‐Whitney *U* test for continuous variables after normality examination in Kolmogorov‐Smirnov test. Analysis of covariance (ANCOVA) for continuous variables and logistic regression for categorical variables were used to compare changes in outcome measures between cilostazol and placebo groups. Because lacune, CMB, and PVS score were not normally distributed, a cutoff of >0 was applied on the presence lacunes and microbleeds, whereas a cutoff of >1 was applied on PVS rating (ranging 0–4) to dichotomize the variables in order to perform logistic regression. Years of education, background hypertension, baseline hippocampal ratio, and CMB were included as covariates for all analyses. Baseline clinical or imaging measures were included as covariates in relevant longitudinal analyses on change in clinical or imaging measurements. Inter‐rater agreement was gauged by the κ statistics. Primary analyses were intention‐to‐treat, defined as all randomized subjects who had taken at least one dose of the study medication. Per‐protocol analyses were performed for subjects who had completed the trial regimen covering the entire study period. We performed efficacy analysis of change in all the outcome measures at the first and second year follow‐up. A two‐sided *p* < 0.05 was considered statistically significant. All statistical analyses were performed using Python 3.7.15.

### 2.12 Secondary analysis

We performed a secondary analysis using a propensity score‐matching model in view of the unbalanced baseline characteristics (years of education, hypertension, baseline hippocampal ratio, CMB) between the two groups despite randomization. In a logistic regression model, we estimated the propensity score of each patient using age, years of education, hypertension, baseline hippocampal ratio, and CMB as covariates subjects with extreme propensity scores were removed (*n* = 28).

## 3 RESULTS

### 3.1 Subjects

**FIGURE 1:**
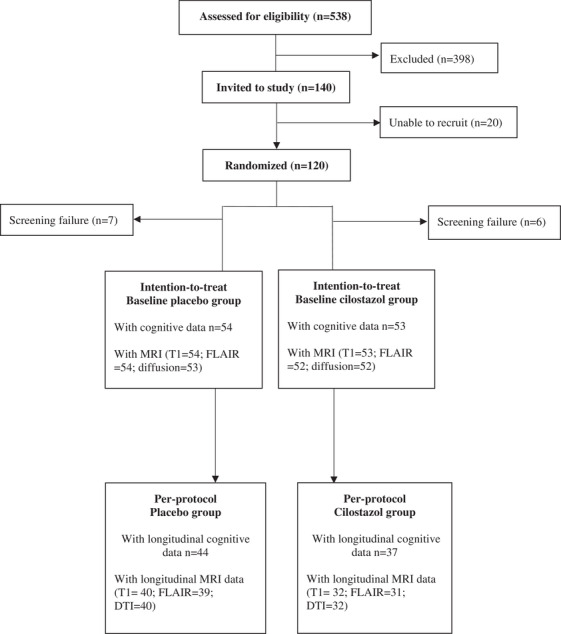
CONSORT diagram

From October 2014 through January 2019, a total of 538 individuals were screened for eligibility; 418 of them did not meet the inclusion criteria. A total of 120 subjects were randomized (*n* = 60 per group). Seven subjects in the treatment group and six subjects in the placebo group withdrew before the first dose of cilostazol or placebo after randomization. Therefore, the intention‐to‐treat sample consisted of 53 subjects in the cilostazol group and 54 subjects in the placebo group. A total of 37 subjects in the cilostazol group and 44 subjects in the placebo group completed the entire trial regimen for per‐protocol analyses (Figure 1).

Overall, the mean age of the subjects was 74.1 ± 4.6 years old; 51 (47.7%) of the subjects were female, 71 (66.4%) had hypertension, 20 (18.7%) had diabetes mellitus, 29 (27.1%) had hyperlipidemia. The placebo group received a shorter duration of education (7.1 ± 4.6 vs 9.4 ± 5.5, *p* = 0.019) and had a higher proportion of hypertensive subjects (75.9% vs 56.6%, *p* = 0.034). Other baseline demographics, medical co‐morbidities, cognitive, gait, and balance performance were similar between the two groups (Table 1). The baseline hippocampal ratio was lower in the cilostazol group (0.5 ± 0.1 vs 0.4 ± 0.1, *p* = 0.012). There were fewer subjects with CMB in the cilostazol group (13.3% vs 34.6%, *p* = 0.010) (Table 2).

**TABLE 1:** Baseline comparison between placebo group and cilostazol group

	Placebo group	Cilostazol group	*p*‐value
**Demographics**			
Age, y; mean ± SD (Placebo: *n* = 54, Cilostazol: *n* = 53)	74.0 ± 4.7	74.0 ± 4.5	0.870
Female, *n*(%) (Placebo: *n* = 54, Cilostazol: *n* = 53)	24 (44.4)	27 (50.9)	0.501
Education, y; mean ± SD (Placebo: *n* = 54, Cilostazol: *n* = 52)	7.1 ± 4.6	9.4 ± 5.5	0.019
**Medical co‐morbidities**			
Hypertension, *n*(%) (Placebo: *n* = 54, Cilostazol: *n* = 53)	41 (75.9)	30 (56.6)	0.034
Diabetes mellitus, *n*(%) (Placebo: *n* = 54, Cilostazol: *n* = 53)	10 (18.5)	10 (18.9)	0.963
Hyperlipidemia, *n*(%) (Placebo: *n* = 54, Cilostazol: *n* = 53)	16 (29.6)	13 (24.5)	0.553
Heart disease, *n*(%) (Placebo: *n* = 54, Cilostazol: *n* = 53)	0 (0%)	0 (0%)	/
Smoking, *n*(%) (Placebo: *n* = 54, Cilostazol: *n* = 53)	5 (9.3)	7 (13.2)	0.518
Drinking, *n*(%) (Placebo: *n* = 54, Cilostazol: *n* = 53)	0 (0%)	0 (0%)	/
**Vascular risk factors**			
Systolic blood pressure; mean ± SD (Placebo: *n* = 52, Cilostazol: *n* = 53)	138.3 ± 17.1	135.2 ± 14.3	0.343
Diastolic blood pressure; mean ± SD (Placebo: *n* = 52, Cilostazol: *n* = 53)	79.8 ± 11.4	78.1 ± 10.3	0.412
Fasting blood glucose level; mean ± SD (Placebo: *n* = 52, Cilostazol: *n* = 53)	5.7 ± 0.9	5.6 ± 1.2	0.175
Glycated haemoglobin A1C level; mean ± SD (Placebo: *n* = 48, Cilostazol: *n* = 52)	6.1 ± 0.7	6.0 ± 0.8	0.609
Cholesterol level; mean ± SD (Placebo: *n* = 53, Cilostazol: *n* = 53)	4.9 ± 1.0	4.9 ± 0.8	0.970
High‐density lipoprotein cholesterol level; mean ± SD (Placebo: *n* = 53, Cilostazol: *n* = 52)	1.5 ± 0.5	1.6 ± 0.5	0.172
Triglyceride level; mean ± SD (Placebo: *n* = 53, Cilostazol: *n* = 52)	1.6 ± 1.7	1.2 ± 0.5	0.285
Low‐density lipoprotein cholesterol level; mean ± SD (Placebo: *n* = 53, Cilostazol: *n* = 52)	2.6 ± 0.7	2.6 ± 0.8	0.962
**Cognition**			
MoCA total score; mean ± SD (Placebo: *n* = 54, Cilostazol: *n* = 52)	21.8 ± 4.0	22.4 ± 4.8	0.320
NINDS 30‐min summary *z*‐score; mean ± SD (Placebo: *n* = 54, Cilostazol: *n* = 53)	0.006 ± 0.629	0.07 ± 0.763	0.248
HKLLT, total learning; mean ± SD (Placebo: *n* = 54, Cilostazol: *n* = 53)	20.3 ± 6.3	19.3 ± 6.3	0.066
Symbol digit modalities test, correct hit; mean ± SD (Placebo: *n* = 54, Cilostazol: *n* = 53)	26.5 ± 11.1	29.7 ± 12.1	0.998
Animal fluency, correct response; mean ± SD (Placebo: *n* = 54, Cilostazol: *n* = 53)	14.8 ± 3.9	17.3 ± 10.6	0.172
**Mood**			
Geriatric Depression Scale Total; mean ± SD (Placebo: *n* = 54, Cilostazol: *n* = 53)	2.0 ± 2.3	2.6 ± 3.2	0.137
**Motor functions**			
8‐min walk test; mean ± SD (Placebo: *n* = 52, Cilostazol: *n* = 52)	8.1 ± 1.6	8.1 ± 2.2	0.731
Single leg stance; mean ± SD (Placebo: *n* = 52, Cilostazol: *n* = 52)	23.1 ± 23.1	20.6 ± 18.4	0.797

Abbreviations: HKLLT, Hong Kong List Learning Test; MoCA, Montreal Cognitive Assessment; NINDS, National Institute of Neurological Diseases and Stroke.

**TABLE 2:** Baseline imaging comparison between placebo group and cilostazol group

	Placebo group	Cilostazol	*p*‐value
Normalized white matter hyperintensity volume, mL; mean ± SD (Placebo: *n* = 53, Cilostazol: *n* = 53)	20.0 ± 15.6	17.3 ± 14.2	0.445
Normalized brain volume, 10^3^ mm^3^; mean ± SD (Placebo: *n* = 54, Cilostazol: *n* = 53)	1353.7 ± 53.1	1347.3 ± 58.7	0.553
Hippocampal ratio; mean ± SD (Placebo: *n* = 53, Cilostazol: *n* = 51)	0.5 ± 0.1	0.4 ± 0.1	0.012
Cerebral microbleeds (>0), *n*(%); Median ± IQR^a^ (Placebo: *n* = 52, Cilostazol: *n* = 53)	18 (34.6%) 1.0 ± 1.0	7 (13.2%) 1.0 ± 0.0	0.010
Lacunes (>0); *n*(%); Median ± IQR^a^ (Placebo: *n* = 53, Cilostazol: *n* = 53)	17 (32.1%) 1.0 ± 1.0	11 (20.8%) 1.0 ± 0.0	0.186
Perivascular space rating (>1) *n*(%); Median ± IQR^b^ (Placebo: *n* = 53, Cilostazol: *n* = 53)	26 (49.1%) 2.0 ± 1.0	23 (43.4%) 2.0 ± 0.0	0.559
Fractional anisotropy; mean ± SD (Placebo: *n* = 53, Cilostazol: *n* = 52)	0.4 ± 0.01	0.4 ± 0.01	0.563
Mean diffusivity, 10^−4^ mm^2^/s; mean ± SD (Placebo: *n* = 53, Cilostazol: *n* = 52)	7.7 ± 0.4	7.6 ± 0.3	0.761

^a^Median ± interquartile range (IQR) was only presented for those with lesions.

^b^Median ± IQR was only presented for PVS score >1.

### 3.2 Imaging outcomes

From the intention‐to‐treat analysis, the difference in WMH volume changes between cilostazol and placebo were not statistically significant (0.3 ± 1.0 mL vs −0.1 ± 0.8 mL, *p* = 0.160). Changes in the normalized brain volume, hippocampal ratio, number of CMBs, lacunes, PVS, and diffusion metrics were also not statistically different between the two groups. Other secondary imaging outcomes are illustrated in Table 3. Per‐protocol analyses of MRI data also did not show statistical significance between the two groups (Table 4).

**TABLE 3:** Changes in MRI measures (baseline vs 2 years)—intention‐to‐treat analysis

	Placebo group	Cilostazol group	*p*‐value
Normalized white matter hyperintensity volume, mL; mean ± SD (Placebo: *n* = 43, Cilostazol: *n* = 35)	−0.1 ± 0.8	0.3 ± 1.0	0.160
Normalized brain volume, mm^3^; mean ± SD (Placebo: *n* = 44, Cilostazol: *n* = 37)	−0.9 ± 0.8	−1.0 ± 1.0	0.848
Hippocampal ratio; mean ± SD (Placebo: *n* = 40, Cilostazol: *n* = 33)	0.0 ± 0.0	0.0 ± 0.0	0.748
Cerebral microbleeds (>0), *n*(%); Median ± IQR^a^ (Placebo: *n* = 41, Cilostazol: *n* = 35)	8 (19.5%) 1.0 ± 0.25	5 (14.3%) 1.0 ± 0.0	0.099
Lacunes (>0), *n*(%); Median ± IQR^a^ (Placebo: *n* = 42, Cilostazol: *n* = 34)	1 (2.4%) −1.0 ± 0.0	2 (5.9%) 0.0 ± 1.0	0.159
Perivascular space count (>0), *n*(%); Median ± IQR^a^ (Placebo: *n* = 43, Cilostazol: *n* = 35)	7 (16.3%) 1.0 ± 0.0	5 (14.3%) 1.0 ± 0.0	0.364
Fractional anisotropy; mean ± SD (Placebo: *n* = 42, Cilostazol: *n* = 36)	−0.002 ± 0.01	−0.003 ± 0.01	0.953
Mean diffusivity, 10^−4^ mm^2^/s; mean ± SD (Placebo: *n* = 42, Cilostazol: *n* = 36)	−0.01 ± 0.2	0.06 ± 0.2	0.462

^a^Median ± interquartile range (IQR) was only presented for those with a change in the lesion measured.

**TABLE 4:** Changes in MRI measures (baseline vs 2 years)—per‐protocol analysis

	Placebo group	Cilostazol group	*p*‐value
Normalized white matter hyperintensity volume, mL; mean ± SD (Placebo: *n* = 39, Cilostazol: *n* = 31)	−0.1 ± 0.9	0.3 ± 1.0	0.129
Normalized brain volume, mm^3^; mean ± SD (Placebo: *n* = 40, Cilostazol: *n* = 32)	−0.9 ± 0.8	−1.0 ± 1.0	0.762
Hippocampal ratio; mean ± SD (Placebo: *n* = 38, Cilostazol: *n* = 31)	0.0 ± 0.0	0.0 ± 0.0	0.782
Cerebral microbleeds (>0), *n*(%); Median ± IQR^a^ (Placebo: *n* = 38, Cilostazol: *n* = 32)	8 (21.1%) 1.0 ± 0.25	5 (15.6%) 1.0 ± 0.0	0.188
Lacunes (>0), *n*(%); Median ± IQR^a^ (Placebo: *n* = 39, Cilostazol: *n* = 32)	1 (2.6%) −1.0 ± 0.0	2 (6.3%) 0.0 ± 1.0	0.324
Perivascular space coun (>0), *n*(%); Median ± IQR^a^ (Placebo: *n* = 40, Cilostazol: *n* = 32)	6 (15%) 1.0 ± 0.0	5 (15.6%) 1.0 ± 0.0	0.575
Fractional anisotropy; mean ± SD (Placebo: *n* = 39, Cilostazol: *n* = 32)	−0.002 ± 0.007	−0.003 ± 0.01	0.727
Mean diffusivity, 10^−4^ mm^2^/s; mean ± SD (Placebo: *n* = 39, Cilostazol: *n* = 32)	0.0 ± 0.2	0.1 ± 0.2	0.251

^a^Median ± interquartile range (IQR) was only presented for those with a change in the lesion measured.

### 3.3 Clinical outcomes

Cognitive outcomes (MoCA, NINDS‐CSN VCI Neuropsychology Protocol 30‐min battery), mood, gait, and stance were not statistically different between the two groups (Table 5). One patient in the placebo group developed a MoCA score of ≤2nd percentile at 2 years (Table S1). Ischemic stroke occurred in one patient (1.9%) in the placebo group and one patient (1.9%) in the cilostazol group (*p* = 1.000). Per‐protocol analysis of clinical outcomes was consistent with the intention‐to‐treat analysis (Table S2).

**TABLE 5:** Changes in cognitive, gait, and mood assessment (baseline vs 2 years)—intention‐to‐treat analysis

	Placebo group	Cilostazol group	*p*‐value
MoCA total score; mean ± SD (Placebo: *n* = 44, Cilostazol: *n* = 37)	0.0 ± 3.2	−0.8 ± 3.1	0.515
NINDS 30 min summary *z*‐score; mean ± SD (Placebo: *n* = 44, Cilostazol: *n* = 37)	0.0 ± 0.4	−0.1 ± 0.5	0.897
HKLLT, total learning; mean ± SD (Placebo: *n* = 44, Cilostazol: *n* = 37)	2.5 ± 5.7	1.5 ± 4.8	0.996
Symbol digit modalities test; mean ± SD (Placebo: *n* = 44, Cilostazol: *n* = 37)	1.1 ± 5.7	−1.4 ± 4.0	0.331
Animal fluency; mean ± SD (Placebo: *n* = 44, Cilostazol: *n* = 37)	−0.3 ± 3.4	−3.0 ± 12.5	0.098
8‐min walk test; mean ± SD (Placebo: *n* = 38, Cilostazol: *n* = 31)	0.7 ± 1.7	1.1 ± 2.0	0.285
Single leg stance; mean ± SD (Placebo: *n* = 37, Cilostazol: *n* = 30)	−7.4 ± 21.7	−3.2 ± 16.7	0.505
Geriatric Depression Scale; mean ± SD (Placebo: *n* = 43, Cilostazol: *n* = 37)	0.3 ± 2.3	−0.2 ± 1.3	0.199

Abbreviations: HKLLT, Hong Kong List Learning Test; MoCA, Montreal Cognitive Assessment; NINDS, National Institute of Neurological Diseases and Stroke; SD, standard deviation.

### 3.4 Within‐group comparisons

Within‐group comparisons of clinical and radiological outcomes are shown in Table S3–S5. Of note, although cilostazol and placebo treatment were not associated with significant within‐group changes in WMH volume, both groups had statistically significant within‐group deterioration in normalized brain volume, hippocampal ratio, and perivascular space rating in the intention‐to‐treat analyses. Within‐group deterioration in fractional anisotropy awas observed in both groups in the per‐protocol analysis, but the deterioration was not statistically significant in the placebo group.

### 3.5 Safety outcomes

Cilostazol use was associated with a significantly higher rate of ankle edema (*p* = 0.013). Palpitation and headache were more common with cilostazol treatment but did not reach statistical significance. Cilostazol use was not associated with increased bleeding, vascular events, or death compared to placebo (Table S6).

### 3.6 Secondary analyses

Forty patients in the cilostazol group and 39 patients in the placebo group remained in the secondary analyses after propensity score matching. Absolute mean standardized differences were < 0.3 across all covariates. No significant differences in baseline characteristics were observed after propensity score matching (Table S7A–D). Similar to the primary analysis, comparisons of all primary and secondary outcomes between cilostazol and placebo groups were not statistically significant.

## 4 DISCUSSION

In this randomized, double‐blind, placebo‐controlled trial, the use of cilostazol did not prevent the progression of WMHs compared to placebo in stroke‐ and dementia‐free subjects with SVD.

The DREAM study is the first clinical trial that recruited stroke‐ and dementia‐free subjects harboring moderate to severe WMH. Because WMH is highly prevalent in the elderly population and a strong predictor of dementia risk,^17^ assessing intervention on the different stages of SVD may inform the appropriate treatment timepoint. Experimental studies have suggested that cilostazol may exert neuroprotection by inducing oligodendrocyte precursor cell maturation, reducing oxidative stress, reducing microglial activation, and enhancing amyloid beta (Aβ) clearance.^35^, ^36^, ^37^ Clinical studies in the prevention of cognitive decline using cilostazol, however, are scarce with mixed results. In a cognitive outcome sub‐study of the PICASSO trial, cilostazol was not associated with better cognitive outcomes compared to aspirin in patients with previous lacunar infarcts, transient ischemic attack, intracerebral hemorrhage, or multiple CMBs.^10^ The LACI‐1 trial suggested that cilostazol use was associated with a reduction in WMHs and a trend toward better cognitive outcomes in patients with clinical lacunar infarcts.^11^ A pilot randomized trial involving patients with co‐existing Alzheimer's disease (AD) and cerebrovascular disease (CVD) suggested that cilostazol treatment may prevent cognitive decline and improve regional cerebral blood flow (CBF).^8^

Our study results implied no significant benefits with cilostazol treatment. Plausible reasons include heterogeneity in study designs and participants. Compared to trials that included patients with clinical strokes,^10^, ^11^ our stroke‐free cohort may be at an earlier stage of SVD. Thus the response to the vasodilatory property of cilostazol may differ based on the following. According to cerebral hemodynamic studies,^38^ an increase in CBF is observed in the earlier phase of cerebral capillary dysfunction. As cerebral capillary dysfunction progresses, a paradoxical attenuation of the CBF is required to relieve the functional shunting from the diseased part of the brain. If the degree of CBF suppression fails to compensate for the capillary dysfunction, excessive functional shunting may cause tissue damage. Therefore, non‐selective vasodilation at earlier stages of SVD by cilostazol may exhibit a different effect on these complex hemodynamic mechanisms and may explain our study findings. However, this finding should be interpreted with caution due to the lower baseline hippocampal ratio in the cilostazol group, although the outcomes remained unchanged with propensity score matching. The direction and magnitude of changes in imaging and cognitive domains within each group were also largely similar. Overall, our findings did not support the use of cilostazol in stroke‐free patients with SVD despite its safety in terms of bleeding, vascular events, and death.

There were several limitations to our study. First, the sample size was relatively small, and thus challenging to avoid type II error. Second, the cilostazol group had a high rate of withdrawal. However, the observations across the intention‐to‐treat and per‐protocol analyses were largely consistent. Third, the lower baseline hippocampal ratio in our treatment group may have confounded the comparisons. Fourth, because the recruitment took place in a community setting involving stroke‐ and dementia‐free subjects, the results of this study may not be generalized to all patients with WMH. Since cilostazol did not worsen WMH progression, in addition to a relatively favorable safety profile and possible benefits in SVD patients with stroke,^11^ further studies should substantiate the effects of cilostazol on different clinical stages of SVD. Fifth, although the literature suggested that WMHs may progress over a 2‐year period in asymptomatic subjects and a portion of our cohort did develop WMH progression,^39^ the average longitudinal within‐group changes of cilostazol and placebo arms were not significant. Ascertaining the therapeutic effect of cilostazol could, therefore, be challenging, as WMH volume in the placebo group did not progress significantly as expected. Nevertheless, both groups exhibited within‐group deterioration in perivascular spaces, brain volume, and diffusion metrics, whereas the degrees of deterioration was not statistically different between cilostazol and placebo. Future studies may adopt a longer follow‐up period, or more sensitive radiological markers as the primary endpoint for patients at lower risk of WMH progression. Finally, our study results remained to be confirmed in other ethnicities due to possible differences in interracial susceptibility to WMHs.^14^

In conclusion, cilostazol treatment did not affect the progression of WMH in stroke‐ and dementia‐free subjects harboring moderate‐to‐severe WMH but demonstrated an acceptable safety profile. Our findings do not support the use of cilostazol in SVD patients without overt clinical symptoms. Future cilostazol trials should address treatment effects on subjects at different clinical stages of SVD.

## CONFLICTS OF INTEREST

Partially funded by a grant from Otsuka Pharmaceutical (H.K.) Ltd. This funding source had no role in the design, execution, analyses, interpretation of the data, or decision to submit results of this study. Bonaventure Y.M. Ip, Bonnie Y.K. Lam, Vincent M.H. Hui, Lisa W.C. Au, Mandy W.T. Liu, Vivian W.Y. Lee, Winnie C.W. Chu, Thomas W. Leung, Ho Ko, and Vincent C.T. Mok declare that they have no potential conflicts of interest that might be relevant to the contents of the manuscript. L. Shi is the director of BrainNow Medical Technology Limited, which assisted with the quantification of hippocampal ratio. Author disclosures are available in the supporting information.

## Supporting information

SUPPORTING INFORMATION

SUPPORTING INFORMATION

SUPPORTING INFORMATION
