# Supplementary material for: Efficacy and safety of cilostazol in decreasing progression of cerebral white matter hyperintensities—A randomized controlled trial
Source: Alzheimers Dement (N Y). 2022 Dec 27;8(1):e12369. doi: 10.1002/trc2.12369 (PMC9793825; doi:10.1002/trc2.12369)
Supplement: Supplementary file 1 — SUPPORTING INFORMATION [file TRC2-8-e12369-s002.docx]

**Figure S1. Age-related white matter changes in the DREAM cohort**

| 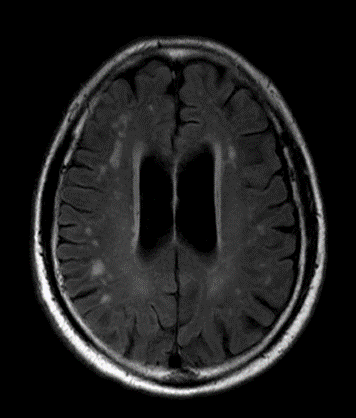(a) | **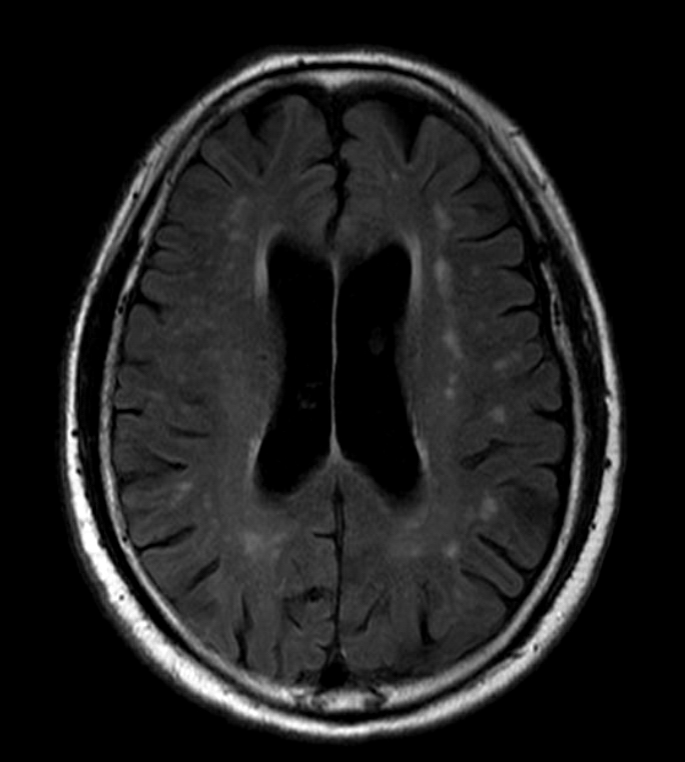**(b) | **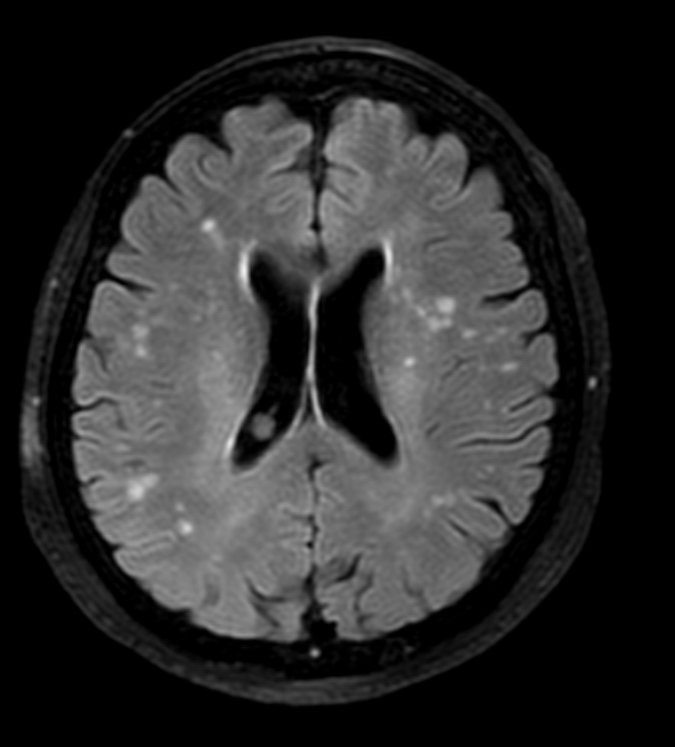** (c) | 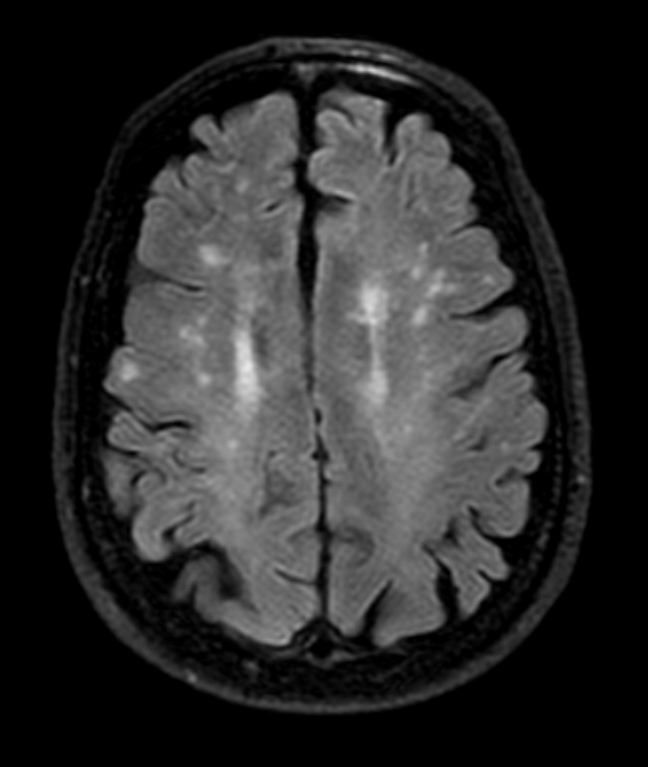 (d) |
| --- | --- | --- | --- |
| **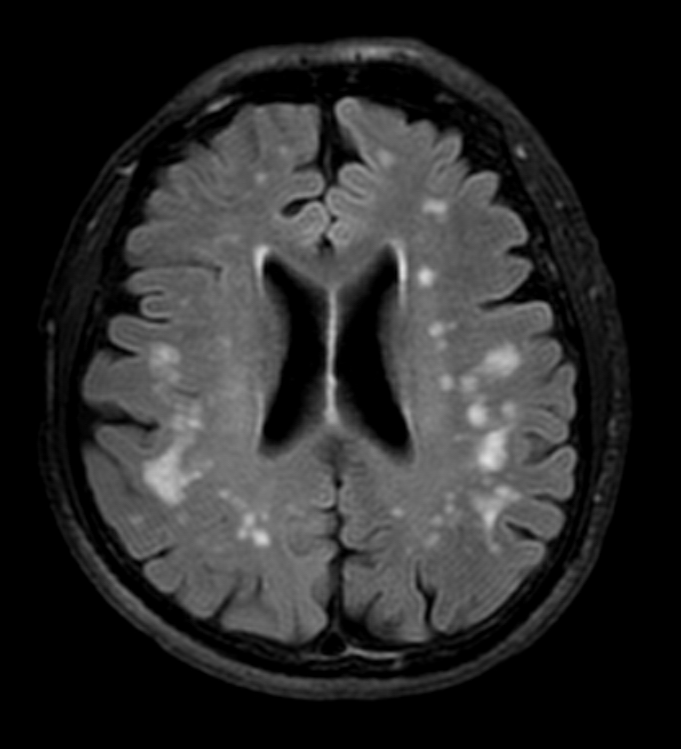**(e) | **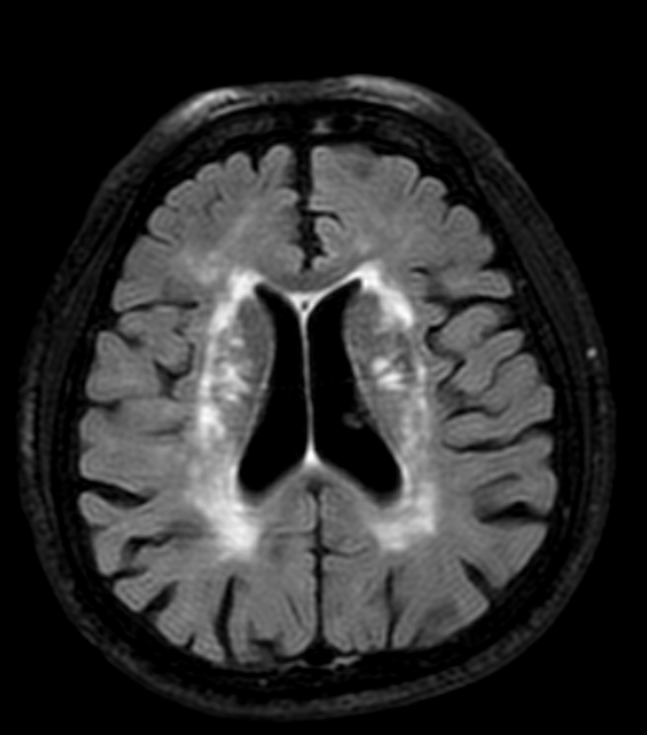** (f) | **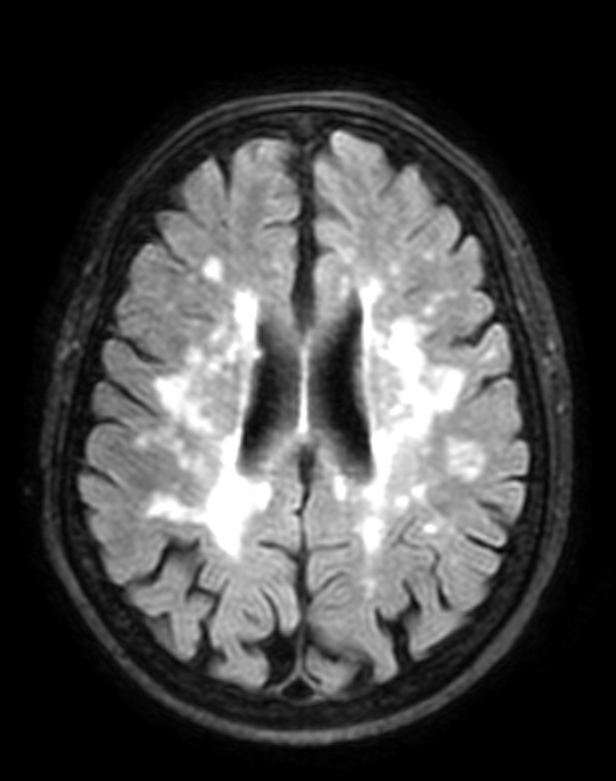** (g) | **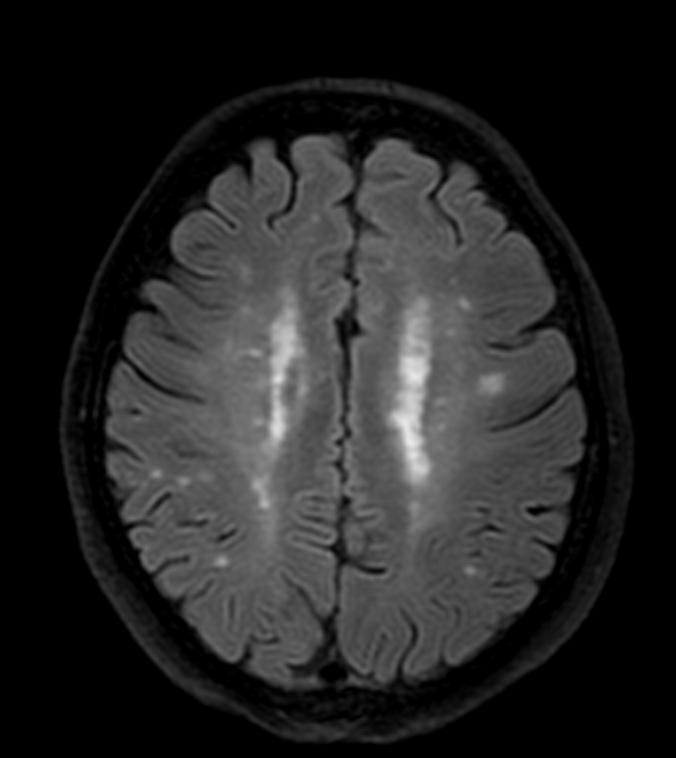** (h) |

Figure S1a-d shows patients with age-related white matter changes (ARWMC) grade 2, note the beginning confluent what matter hyperintensities (WMH). Figure S1e-h shows patients with ARWMC grade 3 with more diffused confluent WMH.

**Table S1 MoCA cut-off at 2^nd^/7^th^/16^th^ percentile**

|  |  | (16^th^, 100^th^] | (7^th^, 16^th^] | (2^nd^, 7^th^] | [1^st^, 2^nd^] |
| --- | --- | --- | --- | --- | --- |
| Placebo  (n=44) | Baseline | 33 | 6 | 5 | 0 |
|  | 2 years | 32 | 6 | 5 | 1 |
| Cilostazol  (n=37) | Baseline | 30 | 5 | 2 | 0 |
|  | 2 years | 30 | 3 | 4 | 0 |

**Table S2. Changes in cognitive, gait, and mood assessment (baseline vs 2 years) – per-protocol analysis**

|  | Placebo group | Cilostazol group | p-value |
| --- | --- | --- | --- |
| MoCA total score; mean ± SD  (Placebo: n=40, Cilostazol: n=32) | 0.0 ± 3.2 | -0.8 ± 3.3 | 0.743 |
| NINDS 30 minutes summary z-score; mean ± SD  (Placebo: n=40, Cilostazol: n=32) | 0.0 ± 0.4 | 0.0 ± 0.4 | 0.695 |
| HKLLT, total learning; mean ± SD (Placebo: n=40, Cilostazol: n=32) | 2.4± 5.6 | 1.9 ± 5.0 | 0.889 |
| Symbol Digit Modalities Test; mean ± SD (Placebo: n=40, Cilostazol: n=32) | 0.9 ± 5.8 | -1.7 ± 4.1 | 0.335 |
| Animal Fluency; mean ± SD  (Placebo: n=40, Cilostazol: n=32) | -0.2 ± 3.5 | -1.1 ± 4.3 | 0.357 |
| 8-minute walk test; mean ± SD  (Placebo: n=37, Cilostazol: n=29) | 0.8 ± 1.7 | 1.2 ± 2.1 | 0.290 |
| Single leg stance; mean ± SD  (Placebo: n=36, Cilostazol: n=28) | 1.9 ± 59.1 | -3.4 ± 17.3 | 0.487 |
| Geriatric Depression Scale; mean ± SD (Placebo: n=40, Cilostazol: n=32) | 0.3 ± 2.4 | -0.2 ± 1.4 | 0.244 |

Abbreviations:

MoCA: Montreal Cognitive Assessment; NINDS: National Institute of Neurological Diseases and Stroke; HKLLT: Hong Kong List Learning Test.

**Table S3 Within-group analyses of brain measurements (over 2 years)**

**Table S3a Intention-to-treat analysis**

| **Intention-to-treat analysis** | | | | | | |
| --- | --- | --- | --- | --- | --- | --- |
|  | Placebo group | | | Cilostazol group | | |
|  | Baseline | 2 years | p-value | Baseline | 2 years | p-value |
| Normalised white matter hyperintensity volume; mL; mean±SD (Placebo: n=43, Cilostazol: n=35) | 19.04±12.32 | 18.92±12.21 | 0.734 | 17.57±8.92 | 17.84±9.23 | 0.219 |
| Normalized brain volume, 10^3^ mm^3^; mean±SD (Placebo: n=44, Cilostazol: n=37) | 1353.62±  54.92 | 1353.62±  54.92 | <0.0001 | 1344.70±  61.72 | 1344.70±  61.72 | <0.0001 |
| Hippocampal ratio; mean±SD (Placebo: n=40, Cilostazol: n=33) | 0.44±0.052 | 0.44±0.055 | 0.0002 | 0.42±0.047 | 0.42±0.050 | 0.0006 |
| Cerebral microbleeds(>0); n(%); *median±IQR (Placebo: n=41, Cilostazol: n=35) | 14 (34.1%)  1±0.75 | 16 (39%)  1±1.25 | 0.157 | 4 (11.4%)  1±1 | 8 (22.9%)  0.5±0.25 | 0.046 |
| Lacunes(>0); n(%); *median±IQR (Placebo: n=42, Cilostazol: n=34) | 14 (33.3%)  1±1 | 13 (31%)  1±1 | 0.317 | 6 (17.6%)  1±0 | 6 (17.6%)  1±0 | 1.0 |
| Perivascular space score(>1) n(%); **median±IQR (Placebo: n=43, Cilostazol: n=35) | 21 (48.8%)  2±0 | 24 (55.8%)  2±1 | <0.0001 | 14 (40%)  2±0 | 16 (45.7%)  2±1 | <0.0001 |
| Fractional anisotropy; mean±SD (Placebo: n=42, Cilostazol: n=36) | 0.396±0.019 | 0.394±0.018 | 0.082 | 0.397±0.015 | 0.394±0.015 | 0.103 |
| Mean diffusivity, 10^-4^ mm^2^/s; mean±SD (Placebo: n=42, Cilostazol: n=36) | 7.61±0.31 | 7.61±0.28 | 0.754 | 7.62±0.25 | 7.68±0.23 | 0.010 |

This table supplemented the within-group differences between baseline and follow-up measures for each group. For continuous variables that are normally distributed, the paired t-test was used. For continuous variables that are not normally distributed, the Wilcoxon signed rank test was used. For binary variables, the McNemar’s test was used (see underlined; median±IQR was presented instead).

**Table S3b Per-protocol analysis**

| **Per-protocol analysis** | | | | | | |
| --- | --- | --- | --- | --- | --- | --- |
|  | Placebo group | | | Cilostazol group | | |
|  | Baseline | 2 years | p-value | Baseline | 2 years | p-value |
| Normalised white matter hyperintensity volume; mL; mean±SD (Placebo: n=39, Cilostazol: n=31) | 19.58±12.53 | 19.45±12.42 | 0.631 | 18.07±9.17 | 18.38±9.50 | 0.098 |
| Normalized brain volume, 10^3^ mm^3^; mean±SD (Placebo: n=40, Cilostazol: n=32) | 1355.05±  58.21 | 1355.04±  58.21 | <0.0001 | 1347.41±  65.30 | 1347.41±  65.30 | <0.0001 |
| Hippocampal ratio; mean±SD (Placebo: n=38, Cilostazol: n=31) | 0.45±0.053 | 0.44±0.056 | 0.0002 | 0.42±0.048 | 0.42±0.051 | 0.0005 |
| Cerebral microbleeds (>0); n(%); *median±IQR (Placebo: n=38, Cilostazol: n=32) | 13 (34.2%)  1±1 | 15 (39.5%)  1±1.5 | 0.157 | 4 (12.5%)  1±1 | 8 (25%)  0.5±0.25 | 0.046 |
| Lacunes (>0); n(%); *median±IQR (Placebo: n=39, Cilostazol: n=32) | 14 (35.9%)  1±1 | 13 (33.3%)  1±1 | 0.317 | 5 (15.6%)  1±0 | 5 (15.6%)  1±0 | 1.0 |
| Perivascular space count (>1) n(%); *median±IQR (Placebo: n=40, Cilostazol: n=32) | 20(50%)  2±0.25 | 22 (55%)  2±1 | 0.157 | 12 (37.5%)  2±0 | 14 (43.8%)  2±0.75 | 0.157 |
| Fractional anisotropy; mean±SD (Placebo: n=39, Cilostazol: n=32) | 0.399±0.019 | 0.396±0.018 | 0.065 | 0.398±0.015 | 0.395±0.015 | 0.043 |
| Mean diffusivity, 10^-4^ mm^2^/s; mean±SD (Placebo: n=39, Cilostazol: n=32) | 7.59±0.31 | 7.59±0.28 | 0.614 | 7.62±0.27 | 7.68±0.24 | 0.010 |

This table supplemented the within-group differences between baseline and follow-up measures for each group. For continuous variables that are normally distributed, the paired t-test was used. For continuous variables that are not normally distributed, the Wilcoxon signed rank test was used. For binary variables, the McNemar’s test was used (see underlined; median±IQR was presented instead).

**Table S4. Within-group analyses of cognitive measurements (over 2 years)**

**Table S4a Intention-to-treat analysis**

| **Intention-to-treat analysis** | | | | | | |
| --- | --- | --- | --- | --- | --- | --- |
|  | Placebo group | | | Cilostazol group | | |
|  | Baseline | 2 years | p-value | Baseline | 2 years | p-value |
| MoCA total score; mean±SD (Placebo: n=44, Cilostazol: n=37) | 21.55±3.85 | 21.42±5.17 | 0.714 | 23.23±4.09 | 22.48±4.48 | 0.221 |
| NINDS 30 minutes summary z-score; mean±SD (Placebo: n=44, Cilostazol: n=37) | 0.01±0.64 | 0.03±0.69 | 0.758 | 0.04±0.72 | 0.01±0.77 | 0.597 |
| HKLLT, total learning; mean±SD (Placebo: n=44, Cilostazol: n=37) | 20.50±6.52 | 22.85±7.38 | 0.015 | 19.58±6.28 | 21.35±7.74 | 0.058 |
| Symbol Digit Modalities Test; mean±SD (Placebo: n=44, Cilostazol: n=37) | 25.74±11.58 | 26.74±12.89 | 0.293 | 31.48±9.97 | 29.74±11.53 | 0.025 |
| Animal Fluency; mean±SD (Placebo: n=44, Cilostazol: n=37) | 15.37±3.77 | 14.95±4.40 | 0.457 | 16.13±4.20 | 14.87±5.25 | 0.112 |
| 8-minute walk test; mean±SD (Placebo: n=38, Cilostazol: n=31) | 7.95±1.51 | 8.74±2.29 | 0.001 | 7.43±1.53 | 8.71±2.82 | 0.001 |
| Single leg stance; mean ± SD (Placebo: n=37, Cilostazol: n=30) | 25.12±25.65 | 15.84±10.43 | 0.050 | 24.30±20.60 | 20.80±1087 | 0.648 |
| Geriatric Depression Scale; mean ± SD (Placebo: n=43, Cilostazol: n=37) | 2.03±2.51 | 2.67±3.03 | 0.275 | 2.11±2.74 | 2.04±3.13 | 0.827 |

This table supplemented the within-group differences between baseline and follow-up measures for each group. For continuous variables that are normally distributed, the paired t-test was used. For continuous variables that are not normally distributed, the Wilcoxon signed rank test was used. For binary variables, the McNemar’s test was used (see underlined; median±IQR was presented instead).

Abbreviations: MoCA: Montreal Cognitive Assessment; NINDS: National Institute of Neurological Diseases and Stroke; HKLLT: Hong Kong List Learning Test.

**Table S4b Per-protocol analysis**

| **Per-protocol analysis** | | | | | | |
| --- | --- | --- | --- | --- | --- | --- |
|  | Placebo group | | | Cilostazol group | | |
|  | Baseline | 2 years | p-value | Baseline | 2 years | p-value |
| MoCA total score; mean±SD (Placebo: n=40, Cilostazol: n=32) | 21.53±3.96 | 21.36±5.19 | 0.663 | 23.23±4.09 | 22.48±4.48 | 0.221 |
| NINDS 30 minutes summary z-score; mean±SD (Placebo: n=40, Cilostazol: n=32) | 0.03±0.64 | 0.03±0.72 | 0.964 | 0.04±0.71 | 0.01±0.77 | 0.597 |
| HKLLT, total learning; mean±SD (Placebo: n=40, Cilostazol: n=32) | 20.5±6.63 | 22.86±7.50 | 0.017 | 19.58±6.28 | 21.4±7.74 | 0.058 |
| Symbol Digit Modalities Test; mean±SD (Placebo: n=40, Cilostazol: n=32) | 26.19±11.72 | 27.06±13.18 | 0.383 | 31.48±9.97 | 29.74±11.53 | 0.025 |
| Animal Fluency; mean±SD (Placebo: n=40, Cilostazol: n=32) | 15.47±3.85 | 15.1±4.41 | 0.538 | 16.13±4.20 | 14.87±5.25 | 0.112 |
| 8-minute walk test; mean±SD (Placebo: n=37, Cilostazol: n=29) | 7.95±1.51 | 8.74±2.29 | 0.001 | 7.43±1.53 | 8.70±2.82 | 0.001 |
| Single leg stance; mean ± SD  (Placebo: n=36, Cilostazol: n=28) | 25.12±25.65 | 15.84±10.43 | 0.050 | 24.30±20.60 | 20.79±10.87 | 0.648 |
| Geriatric Depression Scale; mean ± SD  (Placebo: n=40, Cilostazol: n=32) | 2.03±2.51 | 2.67±3.03 | 0.275 | 2.11±2.74 | 2.04±3.13 | 0.827 |

This table supplemented the within-group differences between baseline and follow-up measures for each group. For continuous variables that are normally distributed, the paired t-test was used. For continuous variables that are not normally distributed, the Wilcoxon signed rank test was used. For binary variables, the McNemar’s test was used (see underlined; median±IQR was presented instead).

Abbreviations: MoCA: Montreal Cognitive Assessment; NINDS: National Institute of Neurological Diseases and Stroke; HKLLT: Hong Kong List Learning Test.

**Table S5. Within-group comparison of baseline & follow-up vascular risk factors**

|  | Placebo group | | | Cilostazol group | | |
| --- | --- | --- | --- | --- | --- | --- |
|  | Baseline | 2 years | P-value | Baseline | 2 years | P-value |
| Smoking n(%);(Placebo: n=54, Cilostazol: n=53) | 5(9.3) | 0(0) | <0.001 | 7(13.2) | 0(0) | <0.001 |
| SBP (mmHg); mean ± SD (Placebo: n=52, Cilostazol: n=53) | 138.1 ± 16.5 | 134.5 ± 16.3 | 0.283 | 136.7±15.8 | 130.2±17.0 | 0.051 |
| DBP (mmHg); mean ± SD (Placebo: n=52, Cilostazol: n=53) | 80.0 ± 10.6 | 78.8 ± 8.6 | 0.539 | 78.0±11.0 | 75.2±10.2 | 0.087 |
| HbA1c (%); mean ± SD (Placebo: n=48, Cilostazol: n=52) | 6.2 ± 0.7 | 6.0 ± 0.7 | 0.189 | 6.1±0.9 | 6.2±1.2 | 0.847 |
| Total cholesterol (mmol/L); mean ± SD (Placebo: n=53, Cilostazol: n=53) | 5.0 ± 1.4 | 4.8±0.9 | 0.094 | 4.9±0.9 | 4.8±0.9 | 0.287 |
| HDL (mmol/L); mean ± SD (Placebo: n=53, Cilostazol: n=52) | 1.5 ± 0.5 | 1.5 ± 0.5 | 0.762 | 1.7±0.5 | 1.8±0.6 | 0.007 |
| TG (mmol/L); mean ± SD (Placebo: n=53, Cilostazol: n=52) | 1.8 ± 2.0 | 1.4 ± 0.6 | 0.071 | 1.3±0.5 | 1.0±0.5 | 0.001 |
| LDL (mmol/L); mean ± SD (Placebo: n=53, Cilostazol: n=52) | 2.6 ± 0.8 | 2.6 ± 0.8 | 0.530 | 2.6±0.9 | 2.5±0.8 | 0.162 |

Abbreviation: SBP: systolic blood pressure; DBP: diastolic blood pressure, HbA1c: glycated hemoglobin A1c; HDL: high-density lipoprotein cholesterol; TG: triglyceride; LDL: low-density lipoprotein cholesterol

**Table S6. Safety outcomes**

|  | Placebo group (n=54) | Cilostazol group (n=53) | p-value |
| --- | --- | --- | --- |
| Adverse events n (%) | 3(5.6) | 12 (22.6) | 0.013 |
| Adverse events leading to treatment discontinuation n (%) | 1 (1.9) | 5 (9.4) | 0.113 |
| Headache n (%) | 0 (0) | 3 (5.7) | 0.118 |
| Ankle edema n (%) | 0 (0) | 6 (11.3) | 0.013 |
| Bleeding n (%) | 1 (1.9) | 1 (1.9) | 1.000 |
| Death n (%) | 1 (1.9) | 0 (0) | 1.000 |
| Vascular events n (%) | 1 (1.9) | 1 (1.9) | 1.000 |
| Palpitation n (%) | 0 (0) | 4 (7.6) | 0.057 |

**Table S7. Comparison between placebo group and cilostazol group after propensity score matching**

**Table S7a Baseline comparison between placebo group and cilostazol group**

|  | Placebo group | Cilostazol group | p-value |
| --- | --- | --- | --- |
| **Demographics** |  |  |  |
| Age, y; mean±SD  (Placebo: n=39, Cilostazol: n=40) | 74.6±4.9 | 73.5±4.4 | 0.322 |
| Female, n(%)  (Placebo: n=39, Cilostazol: n=40) | 17(43.6) | 22(55.0) | 0.311 |
| Education, y; mean±SD  (Placebo: n=39, Cilostazol: n=40) | 7.2±4.8 | 8.5±5.6 | 0.264 |
| **Medical co-morbidities** |  |  |  |
| Hypertension, n(%)  (Placebo: n=39, Cilostazol: n=40) | 30(76.9) | 27(67.5) | 0.350 |
| Diabetes mellitus, n(%)  (Placebo: n=39, Cilostazol: n=40) | 7(17.9) | 7(17.5) | 0.958 |
| Hyperlipidemia, n(%)  (Placebo: n=39, Cilostazol: n=40) | 11(28.2) | 10(25.0) | 0.747 |
| Heart Disease, n(%)  (Placebo: n=39, Cilostazol: n=40) | 0(0%) | 0(0%) | / |
| Smoking, n(%)  (Placebo: n=39, Cilostazol: n=40) | 3(7.7) | 7(17.5) | 0.190 |
| Drinking, n(%)  (Placebo: n=39, Cilostazol: n=40) | 0(0%) | 0(0%) | / |
| **Vascular risk factors** |  |  |  |
| SBP; mean±SD  (Placebo: n=38, Cilostazol: n=40) | 137.6±16.4 | 135.2±13.8 | 0.501 |
| DBP; mean±SD  (Placebo: n=38, Cilostazol: n=40) | 78.4±10.6 | 77.7±10.2 | 0.761 |
| FG level; mean±SD  (Placebo: n=38, Cilostazol: n=40) | 5.6±0.8 | 5.5±0.9 | 0.455 |
| HbA1c level; mean±SD  (Placebo: n=34, Cilostazol: n=39) | 6.1±0.7 | 6.0±0.6 | 0.808 |
| Cholesterol level; mean±SD  (Placebo: n=38, Cilostazol: n=39) | 4.9±1.1 | 4.9±0.8 | 0.873 |
| HDL level; mean±SD  (Placebo: n=38, Cilostazol: n=39) | 1.5±0.6 | 1.7±0.5 | 0.079 |
| Triglyceride level; mean±SD  (Placebo: n=38, Cilostazol: n=39) | 1.7±2.0 | 1.2±0.5 | 0.192 |
| LDL level; mean±SD  (Placebo: n=38, Cilostazol: n=39) | 2.6±0.8 | 2.7±0.8 | 0.964 |
| **Cognition** |  |  |  |
| MoCA total score; mean±SD  (Placebo: n=39, Cilostazol: n=39) | 21.7±4.0 | 22.2±5.1 | 0.289 |
| NINDS 30 minutes summary z-score; mean±SD (Placebo: n=39, Cilostazol: n=40) | 0.0±0.7 | 0.1± 0.8 | 0.692 |
| HKLLT, total learning; mean±SD  (Placebo: n=39, Cilostazol: n=40) | 20.5±6.7 | 19.5±6.3 | 0.530 |
| Symbol digit modalities test, correct hit; mean±SD  (Placebo: n=39, Cilostazol: n=40) | 26.3±11.4 | 29.8±12.8 | 0.201 |
| Animal Fluency, correct response; mean±SD (Placebo: n=39, Cilostazol: n=40) | 15.0±4.0 | 17.5±12.0 | 0.419 |
| **Mood** |  |  |  |
| Geriatric Depression Scale Total; mean±SD (Placebo: n=39, Cilostazol: n=40) | 1.8±1.9 | 3.0±3.3 | 0.192 |
| **Motor functions** |  |  |  |
| 8-minute Walk Test; mean±SD  (Placebo: n=38, Cilostazol: n=39) | 7.8±1.5 | 8.1±2.3 | 0.873 |
| Single Leg Stance; mean±SD  (Placebo: n=38, Cilostazol: n=39) | 25.0±26.0 | 22.4±19.7 | 0.944 |

Abbreviations: SBP: systolic blood pressure; DBP: diastolic blood pressure, HbA1c: glycated hemoglobin A1c; HDL: high-density lipoprotein cholesterol; TG: triglyceride; LDL: low-density lipoprotein cholesterol; MoCA: Montreal Cognitive Assessment; NINDS: National Institute of Neurological Diseases and Stroke; HKLLT: Hong Kong List Learning Test.

**Table S7b Baseline imaging comparison between placebo group and cilostazol group**

|  | Placebo group | Cilostazol | p-value |
| --- | --- | --- | --- |
| Normalised white matter hyperintensity volume; mL; mean±SD  (Placebo: n=39, Cilostazol: n=40) | 20.9±16.8 | 17.1±15.2 | 0.292 |
| Normalized brain volume, 10^3^ mm^3^; mean±SD (Placebo: n=39, Cilostazol: n=40) | 1350.1±50.4 | 1356.0±49.4 | 0.599 |
| Hippocampal ratio; mean±SD  (Placebo: n=39, Cilostazol: n=40) | 0.4±0.0 | 0.4±0.0 | 0.371 |
| Cerebral microbleeds (>0); n(%);  *median±IQR  (Placebo: n=39, Cilostazol: n=40) | 9(23.1%)  1.0±1.0 | 7(17.5%)  1.0±0.0 | 0.537 |
| Lacunes (>0); n(%);  *median±IQR  (Placebo: n=39, Cilostazol: n=40) | 11(28.2%)  1.0±0.75 | 11(27.5%)  1.0±0.0 | 0.944 |
| Perivascular space count (>1) n(%);  **median±IQR  (Placebo: n=38, Cilostazol: n=40) | 19(50.0%)  2.0±0.0 | 19(47.5%)  2.0±0.0 | 0.825 |
| Fractional anisotropy; mean±SD  (Placebo: n=39, Cilostazol: n=39) | 0.4±0.0 | 0.4±0.0 | 0.991 |
| Mean diffusivity, 10^-4^ mm^2^/s; mean±SD (Placebo: n=39, Cilostazol: n=39) | 7.6±0.3 | 7.6±0.2 | 0.594 |

**Table S7c Changes in MRI measures (baseline vs 2 years)- intention-to-treat analysis**

|  | Placebo group | Cilostazol group | p-value |
| --- | --- | --- | --- |
| Normalised white matter hyperintensity volume; mL; mean±SD  (Placebo: n=32, Cilostazol: n=28) | -0.2±1.0 | 0.1±0.5 | 0.141 |
| Normalized brain volume, mm^3^; mean±SD (Placebo: n=32, Cilostazol: n=29) | -0.9±0.8 | -0.8±0.9 | 0.586 |
| Hippocampal ratio; mean±SD  (Placebo: n=30, Cilostazol: n=26) | 0.0±0.0 | 0.0± 0.0 | 0.615 |
| Cerebral microbleeds (>0); n(%);  *median±IQR  (Placebo: n=32, Cilostazol: n=27) | 5(15.6%)  1.0±0.0 | 5(18.5%)  1.0±0.0 | 0.407 |
| Lacunes (>0); n(%);  *median±IQR  (Placebo: n=32, Cilostazol: n=26) | 1(3.1%)  -1.0±0.0 | 2(7.7%)  0.0±1.0 | 0.098 |
| Perivascular space count (>0) n(%);  **median±IQR  (Placebo: n=32, Cilostazol: n=27) | 6(18.8%)  1.0±0.0 | 4(14.8%)  1.0±0.0 | 0.849 |
| Fractional anisotropy; mean±SD  (Placebo: n=32, Cilostazol: n=28) | -0.003±0.001 | -0.002±0.01 | 0.636 |
| Mean diffusivity, 10^-4^ mm^2^/s; mean±SD (Placebo: n=32, Cilostazol: n=28) | 0.0±0.2 | 0.1±0.2 | 0.226 |

**Table 7d Changes in cognitive, gait, and mood assessment (baseline vs 2 years) – intention-to-treat analysis**

|  | Placebo group | Cilostazol group | p-value |
| --- | --- | --- | --- |
| MoCA total score; mean±SD  (Placebo: n=32, Cilostazol: n=29) | 0.0±3.4 | -0.6±3.1 | 0.288 |
| NINDS 30 minutes summary z-score; mean±SD  (Placebo: n=32, Cilostazol: n=29) | 0.0±0.4 | -0.1±0.5 | 0.445 |
| HKLLT, total learning; mean±SD  (Placebo: n=32, Cilostazol: n=29) | 1.9±5.8 | 1.2±5.2 | 0.638 |
| Symbol Digit Modalities Test; mean±SD (Placebo: n=32, Cilostazol: n=29) | 0.4±6.2 | -0.9±3.6 | 0.349 |
| Animal Fluency; mean±SD  (Placebo: n=32, Cilostazol: n=29) | 0.2±3.4 | -3.4±14.0 | 0.208 |
| 8-minute walk test; mean±SD  (Placebo: n=28, Cilostazol: n=23) | 0.9±1.8 | 1.4±1.9 | 0.311 |
| Single leg stance; mean±SD  (Placebo: n=27, Cilostazol: n=22) | -9.9±23.3 | -5.2±18.5 | 0.446 |
| Geriatric Depression Scale; mean±SD  (Placebo: n=32, Cilostazol: n=29) | 0.7±2.4 | -0.1±1.4 | 0.142 |

Abbreviations: MoCA: Montreal Cognitive Assessment; NINDS: National Institute of Neurological Diseases and Stroke; HKLLT: Hong Kong List Learning Test.
