# Supplementary material for: Efficacy and safety of cilostazol in decreasing progression of cerebral white matter hyperintensities—A randomized controlled trial
Source: Alzheimers Dement (N Y). 2022 Dec 27;8(1):e12369. doi: 10.1002/trc2.12369 (PMC9793825; doi:10.1002/trc2.12369)
Supplement: Supplementary file 2 — SUPPORTING INFORMATION [file TRC2-8-e12369-s003.docx]

**Table S6. Safety outcomes**

|  | Placebo group (n=54) | Cilostazol group (n=53) | p-value |
| --- | --- | --- | --- |
| Adverse events n (%) | 3(5.6) | 15 (28.3) | 0.002 |
| Adverse events leading to treatment discontinuation n (%) | 1 (1.9) | 7 (13.2) | 0.032 |
| Headache n (%) | 0 (0) | 3 (5.7) | 0.118 |
| Ankle edema n (%) | 0 (0) | 6 (11.3) | 0.013 |
| Bleeding n (%) | 1 (1.9) | 1 (1.9) | 1.000 |
| Death n (%) | 1 (1.9) | 0 (0) | 1.000 |
| Vascular events n (%) | 1 (1.9) | 1 (1.9) | 1.000 |
| Palpitation n (%) | 0 (0) | 4 (7.6) | 0.057 |
